# The Dysphagia of Tuberculous Laryngitis

**Published:** 1908-02-15

**Authors:** 


### Laryngology and Rhinology.

### THE DYSPHAGIA OF TUBERCULOUS LARYNGITIS.

The treatment of painful deglutition in cases of tuberculous laryngitis should, from a practical point of view, be considered quite apart from the question of the curative treatment of this disease: for when there is decided dysphagia the affection is usually so far advanced that no cure can be expected. In fact, tuberculous laryngitis may be practically divided into two classes; that in which there is a hope of obtaining improvement by local treatment, and that in which there is none. In the latter the treatment should be carried out on purely symptomatic lines, and, if severe symptoms be absent, only mild measures such as spays, lozenges or inhalations should be employed. But in phthisis, where ample feeding is so important, even a mild degree of dysphagia becomes a symptom of the gravest significance, and results in the rapid downward progress of the patient; in a later stage, if this symptom be not relieved, swallowing becomes almost impossible, and semi-starvation rapidly brings about a fatal termination.

Difficulty in swallowing is of three kinds, all of which are included loosely under the term "dysphagia": painful deglutition, obstruction, and the entrance of food into the larynx. The latter is not very common, but is a complication extremely difficult to combat; it is caused by imperfect closure of the upper aperture of the larynx, and is due to stiffness and rigidity from infiltration rather than to loss of substance from ulceration. True obstructive dysphagia, of a high-grade, such as occurs in œsophageal cancer, is also rare, but results occasionally from extreme swelling of the epiglottis or arytenoids. On the other hand, painful deglutition, or odynphagia is unfortunately very common, and it is this distressing symptom which renders laryngeal tuberculosis such an important and such a terrible disease. It is found in disease of any part of the upper aperture of the larynx; when the epiglottis is affected the difficulty is chiefly with solid food, but in cases of arytenoid infiltration it is often most marked when swallowing fluids; for a bolus of soft food partially protects the tender arytenoid from compression by the contracting pharyngeal muscles. Some anomalies are often met with; thus with many patients the chief difficulty is on swallowing meat, though they can take other and harder food with ease. Milk is frequently peculiarly troublesome, and seems to remain about the throat and cause much irritation. In general, semi-solids are swallowed better than liquids or hard substances. Fluids should therefore be thickened; custards, junkets, jellies, raw eggs and oysters are usually well taken. The latter have the advantage that a rather large mass can be gulped down at one time, which, to patients with dysphagia, is easier than sipping small quantities. Wolfenden's method of feeding is founded on this peculiarity, and is often very helpful; the patient lies prone on a sofa or in bed, with the head projecting over the edge, and sucks up fluid food through a tube or straws from a vessel placed some six inches below his level. Another method which sometimes affords assistance is as follows: The patient sits, and an attendant, standing behind, makes firm pressure along the sides of the neck with the palmar surface of his hands and fingers during the act of swallowing.

In all cases where dysphagia is considerable the exhibition of local anodynes becomes necessary. A strong warning must be given against the early or hasty use of morphine or cocaine; they both quickly lose their potency, the dose has to be rapidly increased, and they have a most disastrous effect on the appetite and general health. They should, therefore, be reserved for the last stages of severe cases, when all other remedies have failed. Morphine should be given as an insufflation in doses of $\frac{1}{8}$ to $\frac{1}{2}$ grain diluted with two grains of starch or bismuth; cocaine is best given as a spray in doses of a few minims of a 5 per cent. solution. The former should be used half an hour and the latter five minutes before eating, and both must be administered only by the doctor or a well-trained nurse. By far the best anodyne for general use in these cases is orthoform; this is an almost insoluble powder, and may be used as an insufflation in doses of 3 to 5 grains; the anæsthetic effect takes nearly an hour to develop fully, and lasts for a long time, often for twelve hours. It is not toxic, and may be entrusted to the patient, and applied by the method of "auto-insufflation"; the patient is provided with a straight glass tube 5 inches long, one end is pushed into the powder and picks up about 3 grains, the other end is then put far back into the mouth, and a sharp inspiration draws a large proportion of the dose into the larynx; the tube must be washed and thoroughly dried before being used again. Anæsthesin is a similar drug, which may be employed in the same way; in general it appears to be less valuable than orthoform, but it sometimes acts when the latter fails.

When, however, the dysphagia is severe, all these methods give but a small modicum of relief; such severe dysphagia is usually found when the arytenoids or epiglottis are much swollen and infiltrated. In these cases removal of the diseased and tender epiglottis, or the punching out of even a small piece from the swollen arytenoid, is a most effective method of relieving this distressing symptom. This result appears to be largely due to the relief of tension, for, after clipping a small piece from the arytenoid region, the swelling of the entire upper aperture will usually diminish to a quite remarkable extent. If a good pattern of punch-forceps be used, the operation can be done very rapidly and painlessly after the application of cocaine. There is so little disturbance or shock that it may be performed on any patient strong enough to sit up, for it is no little matter to allow him to end his last few days in comparative comfort. But the operation should only be done when dysphagia is marked, and not when there is merely discomfort, for otherwise disappointment will be caused.

